# Supplementary material for: Artificial cellulosic leaf with adjustable enzymatic CO2 sequestration capability
Source: Nat Commun. 2024 Jun 8;15:4898. doi: 10.1038/s41467-024-49320-y (PMC11162438; doi:10.1038/s41467-024-49320-y)
Supplement: Supplementary file 1 — Supplementary Information [file 41467_2024_49320_MOESM1_ESM.pdf]

Supplementary Information

Artificial cellulosic leaf with adjustable enzymatic CO_2 sequestration capability

Xing Zhu[1,a,b,*], Chenxi Du[1,a,b], Bo Gao[d] and Bin He[a,c,*]

[a] College of Bioresources Chemical and Materials Engineering, Shaanxi University of Science & Technology, Xi'an, 710021, China

[b] Institute of Biomass & Functional Materials, Shaanxi University of Science & Technology, Xi'an, 710021, China

[c] Key Laboratory of Paper Based Functional Materials, Shaanxi University of Science & Technology, Xi'an, 710021, China

[d] School of Chemical Engineering, Northwest University, Xi'an, 710127, China

[1] These authors contributed equally: Xing Zhu, Chenxi Du

[*] Corresponding author. E-mail: zhuxing@sust.edu.cn; prof.hebin@sust.edu.cn

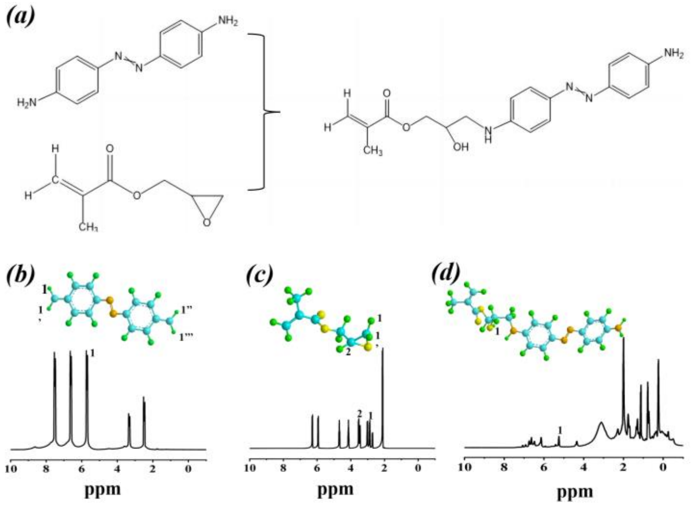

Supplementary Figure 1. Reaction mechanism of GMA and 4,4-AZO. (a) Synthesis mechanism of GMA/4,4'-AZO; (b) ^1H NMR spectra of 4,4'-AZO; (c) GMA; and (d) GMA/4,4'-AZO

The synthesis pathway of GMA/4,4'-AZO is shown in Supplementary Figure 1(a). One of the 5.80 ppm signals in Supplementary Figure 1(b) corresponds to the amino proton peak in 4,4'-AZO. One of the 2.51-3.02 ppm signals in Supplementary Figure 1(c) corresponds to the epoxy-based proton peak in GMA, and the 3.15-3.63 ppm signals correspond to the -CH proton peak in GMA.

In the ^1H NMR spectrum of GMA/4,4'-AZO in Supplementary Figure 1(d), the disappearance of the characteristic amino proton peak in 4,4'-AZO and the nascent hydroxyl proton peak (5.2 ppm) in GMA/4,4'-AZO can be observed, which suggests that 4,4'-AZO's amino group was consumed through an epoxy ring-opening addition reaction with GMA. Thus, in this study, GMA/4,4'-AZO was successfully synthesized using the epoxy ring-opening addition reaction between 4,4'-AZO and GMA.

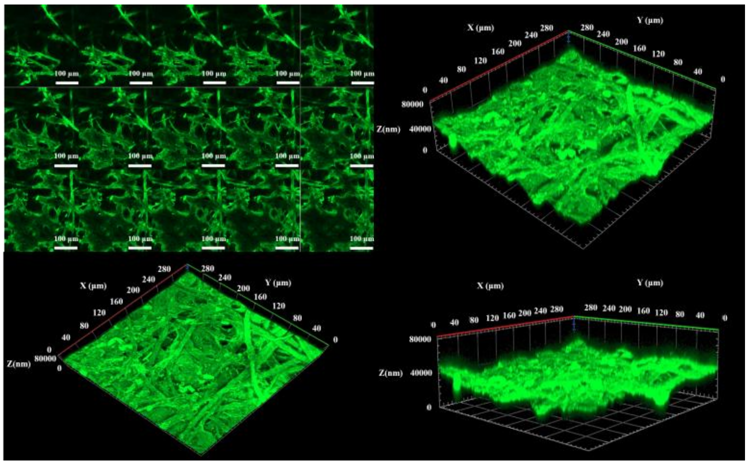

Supplementary Figure 2. Three-dimensional structure of EcoLeaf. 3D tomograms of EcoLeaf and the 3D maps from different top-down view angles under LCSM (0°, 60°, 90°)

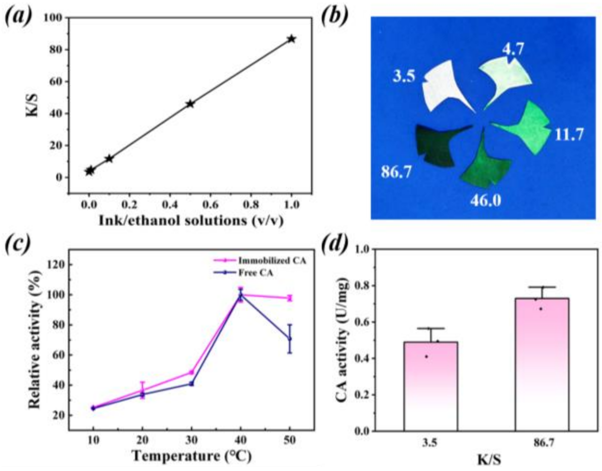

Supplementary Figure 3. Photothermal conversion characteristics of EcoLeaf. (a) Variation of K/S with ink/ethanol solution; (b) Artificial leaves with different K/S values; (c) Optimal carbon fixation temperatures of CA in EcoLeaf and free CA (Parallel experiments with three sets of identical samples) (n=3 independent experiments, data are presented as mean values +/- SEM); (d) Enzyme activities of EcoLeaf with K/S values of 3.5 and 86.7, respectively, after irradiation for 3 mins at an initial temperature of 30 °C and a visible light irradiance of 4.5×10^4

Lux. (n=3 independent experiments)

As shown in Supplementary Figure 3(b), the optimal catalytic temperature of CA encapsulated by EcoLeaf is consistent with that of free CA, which is due to the fact that the encapsulation and immobilization can maximize the maintenance of the protein conformation of CA and prevent the enzyme protein disassembly caused by the temperature change.

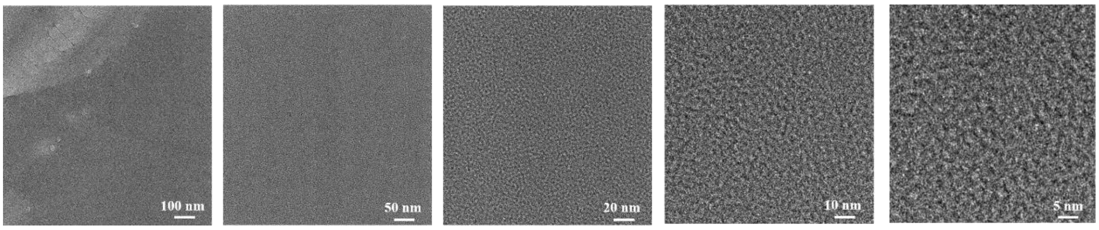

Supplementary Figure 4. The shape of three-dimensional mesh. High-resolution transmission electron micrograph (TEM) of EcoLeaf (scale: 5-100 nm)

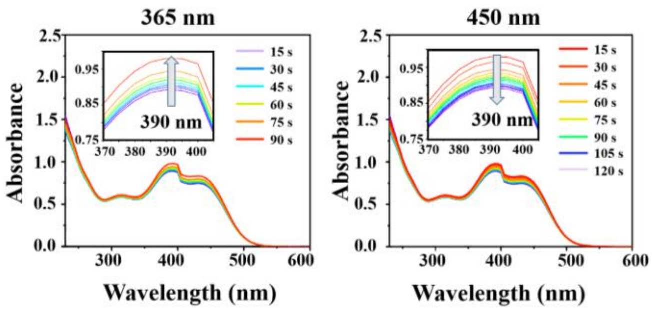

Supplementary Figure 5. Photoresponse properties. Photoresponse properties of GMA/4,4'-AZO solution under (a) 365 nm UV light and (b) 450 nm visible light excitation

From Supplementary Figure 5 (a–b), after irradiating the GMA/4,4'-AZO solution with a 450 nm visible light source (40 mW/cm^2) and a 365 nm UV light source (40 mW/cm^2), the absorption peaks at 390 nm appeared to be increasing and decreasing with the increase in illumination time, respectively. This indicates that GMA/4,4'-AZO still possesses the photoresponsive properties of AZO.

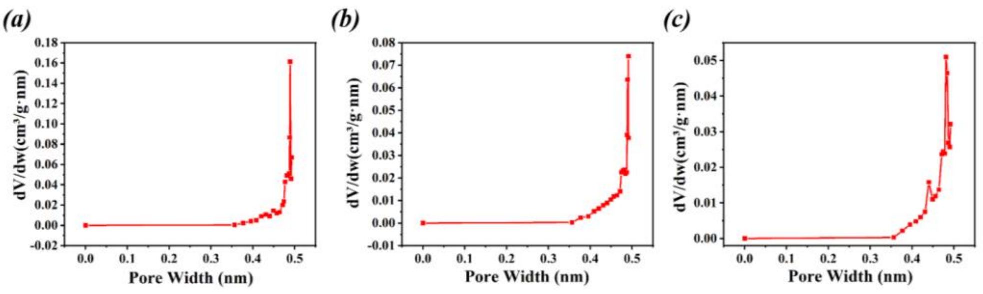

Supplementary Figure 6. Change rule of pore diameter. Pore size distribution curves of

EcoLeaf by one round of irradiation (a) 450 nm, (b) 365 nm, and (c) 450 nm

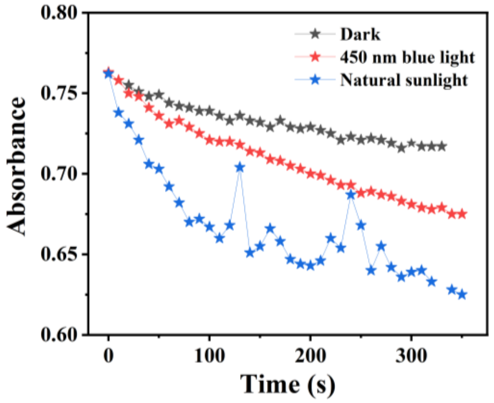

Supplementary Figure 7. Photoacoustic efficiency of EcoLeaf. Changes in absorbance (pore properties) of EcoLeaf under darkness, 450 nm blue light, and natural sunlight after irradiation for 20 min at 365 nm UV light

In practice, natural sunlight contains both 365 nm UV and 450 nm blue light. Therefore, the stomatal expansion and contraction of EcoLeaf under sunlight is critical. As shown in Supplementary Figure 7, when EcoLeaf was irradiated under UV light (365 nm, 20.3 mW/cm^2) for 20 min and then transferred to darkness, blue light (450 nm, 19.1 mW/cm^2), and natural sunlight (27.5–28.8 mW/cm^2), respectively, the absorbance decreased, and the rate of the decrease increased in order. Among them, the slow expansion of stomata in the dark environment was due to the spontaneous transition of GMA/4,4'-AZO from the *cis* form of the substable state to the *trans* form of the steady state (Fiure 4(a)). It can be seen that blue light is able to accelerate the process, and thus the blue light group possesses a higher rate of decrease. The sudden increase in this group was mainly due to the fluctuation of natural light, but the overall decreasing trend was still able to remain stable. In addition, the expansion and contraction of GMA/4,4'-AZO stomata are directly related to light intensity. The greater the intensity of sunlight, the greater the stomatal expansion. Of the solar radiation that reaches the earth's surface from the sun, visible light accounts for about 40% of the total solar radiation, infrared light for about 50%, and ultraviolet light for only about 10%. Since visible light is more intense, the mesh of EcoLeaf tends to expand under natural sunlight. A comparison of the red and blue lines in Supplementary Figure 7 shows that the natural light group has a more significant effect on stomatal expansion because it possesses a stronger light intensity than the blue light group. This demonstrates the dependence of stomatal expansion and contraction on light intensity.

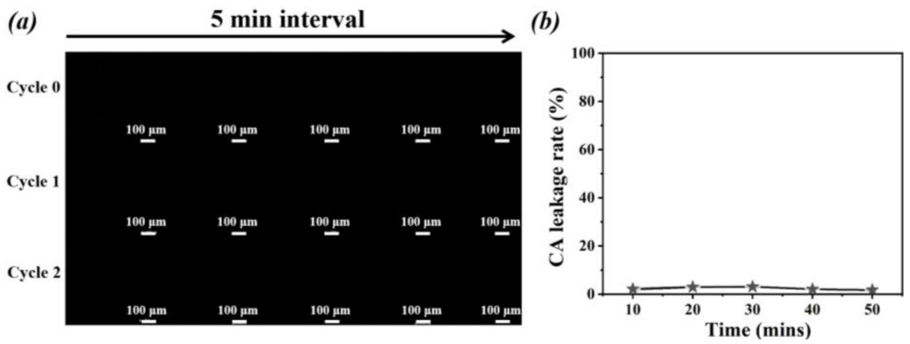

Supplementary Figure 8. Enzyme leakage of EcoLeaf. (a)Aqueous solutions attached to petioles of EcoLeaf containing FITC-CA were observed under LSCM (time interval: 5 min); (b)The CA leakage rate in the aqueous solution (attached to the petioles of EcoLeaf) stained by G-250 was measured by a UV-*vis* spectrophotometer (time interval: 10 min) (Parallel experiments with three sets of identical samples)

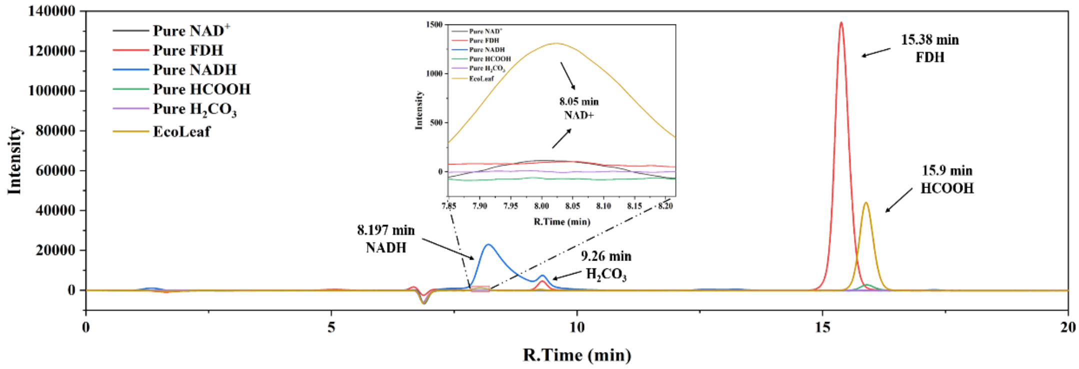

Supplementary Figure 9. Functional designability of EcoLeaf. Chromatograms of pure NADH, NAD^+, H_2CO_3, FDH, HCOOH dissolved in deionized water and the generating solution of the EcoLeaf containing FDH, NADH. Mobile phase H_2SO_4

To verify the scalability of the EcoLeaf bionic system, in this study, the carbon capture core organ CA was replaced with formate dehydrogenase (FDH), which is capable of converting CO_2 to formic acid (HCOOH), and its reducing coenzyme nicotinamide adenine dinucleotide (NADH). The substances involved in the reaction system (pure NADH, NAD^+, H_2CO_3, FDH, and HCOOH dissolved in deionized water) and the solid carbon-generating solution of the EcoLeaf were characterized by high-performance liquid permeation chromatography. As shown in Supplementary Figure 9, pure NADH, NAD^+, H_2CO_3, FDH, and HCOOH peaked at 8.197, 8.05, 9.26, 15.38, and 15.9 min, respectively, and the solid-carbon generating solution of EcoLeaf peaked at 8.05 and 15.9 min, respectively. This demonstrates that CO_2 can be successfully converted to HCOOH by replacing CA with FDH and NADH in the EcoLeaf bionic system. This

demonstrates the high degree of designability of the EcoLeaf system.

Supplementary Table 1. Emerging CO_2 conversion systems with high light energy conversion efficiency

**Raw materials**	**Synthesis method and reaction conditions**	**Light energy usage and CO_2 conversion performance($mol \cdot J^{-1} \cdot g^{-1}$)**	**Cost ($/g)**	**Potential environmental impacts**	**Ref.**
EcoLeaf	Visible light induced graft polymerization	17.3	3.5	Soil degradable non-toxicity	-
Mn-MIL-88A	Hydrothermal treatment (65 °C/12 h)	0.14	-	Airborne particulate matter	1
g-C_3N_4	One-pot thermal reaction (550 °C/4 h)	0.2	3116.7	Excessive energy consumption Increased greenhouse effect	2
Zn-MIL-88A	Hydrothermal treatment (65 °C/12 h)	0.36	-	Airborne particulate matter	1
PVK|FDH	Spin coating and annealing (300 °C)	0.44	-	-	3
$Cu_{27}Pd_{73}$||PVK|$Cu_{91}In_9$		0.76	-	Heavy metal pollution	3
PVK|CoPL		0.95	-	Water pollution	3
$Cs_3Sb_2I_9$	Vacuum in-situ crystallization (120 °C/20 min)	1.67	9.8	Water and soil pollution	4
$CsPbBr_3$/BP	LARP; Self-assembly(-)	2.32	39.9	Heavy metal pollution	5
$CsPbBr_3$/USGO/a-Fe_2O_3	Hot injection and Self-assembly (180 °C/12 h)	2.66	-	Heavy metal pollution	6
$CsPbBr_3$	Facet manipulation (120 °C/1h, 220°C/15 h)	2.76	20.9	Heavy metal pollution	7
$CsPbBr_3$/CTF-1-Ni	Hot injection (120 °C/1.5 h, 100 °C/24 h); Self-assembly	3.11	3.3	Heavy metal pollution	8
Ni-doped $CsPbBr_3$/Bi_3O_4Br	Hot injection (100 °C/1.5 h); Self-assembly	4.14	23.3	Heavy metal pollution	9
T-$SrTiO_3$/$CsPbBr_3$	Hot injection(-);	4.33	4.7	Heavy metal	10

	In-situ growth			pollution	
$FAPbBr_3/Ti_3C_2$	Hot injection(-); Interfacial interaction	4.39	3.0	Heavy metal pollution	11
$Co_{1\%}@CsPbBr_3/Cs_4PbBr_6$	LARP(-)	4.4	2.9	Heavy metal pollution	12
$Co/Co–Al_2O_3$	Calcine (150 °C/24 h, 500 °C/8 h, 700 °C/1 h)	4.42	1.5	Ecotoxicity Excessive energy consumption Increased greenhouse effect	13
$Cs_3Bi_2Br_9$	Hot injection (-)	4.59	-	Soil and water pollution	14
Au rod and copper-palladium (CuPd) alloy shell	Epitaxial growth method (-)	4.95	226.9	Heavy metal pollution	15
$CsPbBr_3$-$SOBr_2$/g-C_3N_4	Hot injection(120 °C/30 min, 150 °C/1h, 500 °C/2 h); Self-assembly	6.84	37.1	Water pollution	16
$Pd@Nb_2O_5$	Microwave-assisted reaction(150 °C/ 20 min)	7.2	194.4	Heavy metal pollution	17
$CsPbBr_3$-Ni(tpy)	Hot injection(100 °C/1 h); Ligand exchange; Self-assembly	15.52	3.3	Water pollution	18
$CsPbBr_3/PbSe$	Hot injection(100 °C/1 h); Self-assembly	19.97	8.2	Soil and water pollution	19
Ternary g-$C_3N_4/TiO_2/Ti_3AlC_2$ 2D/0D/2D composite	Sol-gel method(500 °C/4h, 550 °C/4h)	75.73	19.1	Microplastic pollution Excessive energy consumption Increased greenhouse effect	20
Rh/Al nanoantenna batch	One-pot thermal reaction(160 °C/4 h)	175.22	1634.6	Soil and water pollution	21

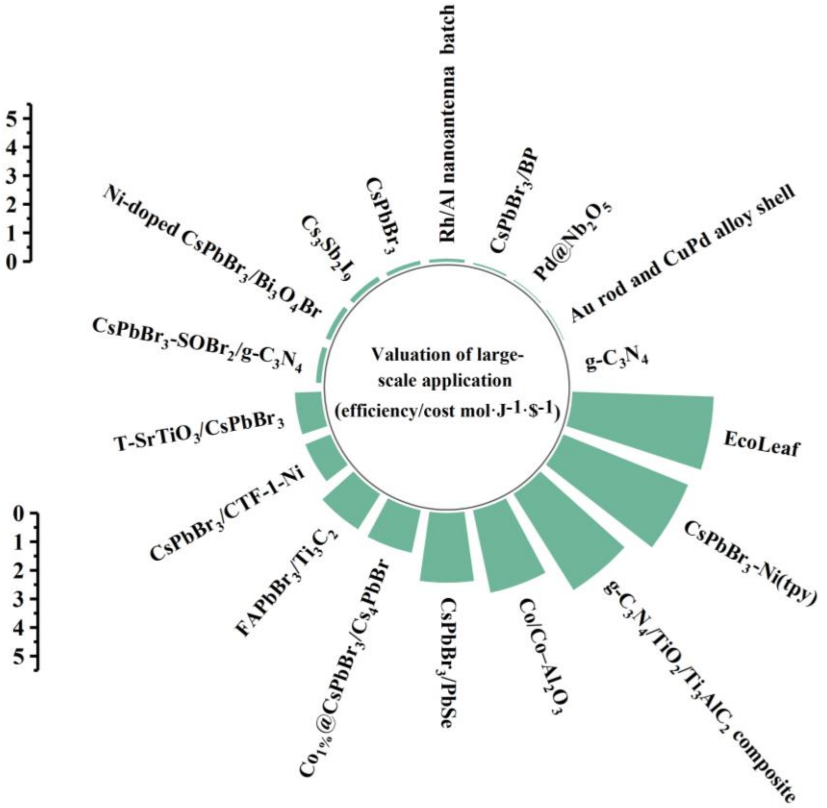

Supplementary Figure 10. The practical application value of EcoLeaf. The prospect of large-scale application (valuation of large-scale application) of the materials through the efficiency/cost of light energy utilization (mol·J^{-1}·\$$^{-1}$) of different photofunctional CO_2 conversion systems

Supplementary References

1. Ojha N, Kumar S. Tri-phase photocatalysis for CO_2 reduction and N_2 fixation with efficient electron transfer on a hydrophilic surface of transition-metal-doped MIL-88A (Fe). *Appl Catal B-Environ* **292**, 120166 (2021).
2. Kamal Hussien M*, et al.* Metal-free four-in-one modification of g-C_3N_4 for superior photocatalytic CO_2 reduction and H_2 evolution. *Chem Eng J* **430**, 132853 (2022).
3. Bhattacharjee S*, et al.* Photoelectrochemical CO_2-to-fuel conversion with simultaneous plastic reforming. *Nat Synth* **2**, 182-192 (2023).
4. Wang Y, Zhou Q, Zhu Y, Xu D. High efficiency reduction of CO_2 to CO and CH_4 via photothermal synergistic catalysis of lead-free perovskite $Cs_3Sb_2I_9$. *Appl Catal B-Environ* **294**, (2021).
5. Wang X*, et al.* Immobilizing perovskite $CsPbBr_3$ nanocrystals on Black phosphorus nanosheets for boosting charge separation and photocatalytic CO_2 reduction. *Appl Catal B-Environ* **277**, 119230 (2020).

6. Mu YF, Zhang W, Dong GX, Su K, Zhang M, Lu TB. Ultrathin and Small-Size Graphene Oxide as an Electron Mediator for Perovskite-Based Z-Scheme System to Significantly Enhance Photocatalytic CO_2 Reduction. *Small* **16**, (2020).

7. Bera S, Shyamal S, Pradhan N. Chemically Spiraling $CsPbBr_3$ Perovskite Nanorods. *J Am Chem Soc* **143**, 14895-14906 (2021).

8. Wang Q*, et al.* Coupling $CsPbBr_3$ Quantum Dots with Covalent Triazine Frameworks for Visible-Light-Driven CO_2 Reduction. *ChemSusChem* **14**, 1131-1139 (2021).

9. Wang X, Wang Z, Li Y, Wang J, Zhang G. Efficient photocatalytic CO_2 conversion over 2D/2D Ni-doped $CsPbBr_3$/Bi_3O_4Br Z-scheme heterojunction: Critical role of Ni doping, boosted charge separation and mechanism study. *Appl Catal B-Environ* **319**, (2022).

10. Yuan S-X, Su K, Feng Y-X, Zhang M, Lu T-B. Lattice-matched in-situ construction of 2D/2D T-$SrTiO_3$/$CsPbBr_3$ heterostructure for efficient photocatalysis of CO_2 reduction. *Chinese Chem Lett* **34**, (2023).

11. Que M*, et al.* Anchoring of Formamidinium Lead Bromide Quantum Dots on Ti_3C_2 Nanosheets for Efficient Photocatalytic Reduction of CO_2. *ACS Appl Mater& Interfaces* **13**, 6180-6187 (2021).

12. Dong G-X, Zhang W, Mu Y-F, Su K, Zhang M, Lu T-B. A halide perovskite as a catalyst to simultaneously achieve efficient photocatalytic CO_2 reduction and methanol oxidation. *Chem Commun* **56**, 4664-4667 (2020).

13. Wu S*, et al.* High light-to-fuel efficiency and CO_2 reduction rates achieved on a unique nanocomposite of Co/Co doped Al_2O_3 nanosheets with UV-vis-IR irradiation. *Energ & Environ Sci* **12**, 2581-2590 (2019).

14. Sheng J*, et al.* Identification of Halogen-Associated Active Sites on Bismuth-Based Perovskite Quantum Dots for Efficient and Selective CO_2-to-CO Photoreduction. *ACS Nano* **14**, 13103-13114 (2020).

15. Hu C*, et al.* Near-infrared-featured broadband CO_2 reduction with water to hydrocarbons by surface plasmon. *Nat Commun* **14**, (2023).

16. Zheng Q*, et al.* Surface Halogen Compensation on $CsPbBr_3$ Nanocrystals with $SOBr_2$ for Photocatalytic CO_2 Reduction. *ACS Mater Lett* **4**, 1638-1645 (2022).

17. Jia J*, et al.* Visible and Near-Infrared Photothermal Catalyzed Hydrogenation of Gaseous CO_2 over Nanostructured Pd@Nb_2O_5. *Adv Sci* **3**, (2016).

18. Chen Z*, et al.* Boosting Photocatalytic CO_2 Reduction on $CsPbBr_3$ Perovskite Nanocrystals by Immobilizing Metal Complexes. *Chem Mater* **32**, 1517-1525 (2020).

19. Zhang G*, et al.* Interfacial Engineering of Semicoherent Interface at Purified $CsPbBr_3$

Quantum Dots/2D-PbSe for Optimal CO_2 Photoreduction Performance. *ACS Appl Mater Interfaces* **14**, 44909-44921 (2022).

20. Tahir M, Tahir B. Constructing S-scheme 2D/0D g-C_3N_4/TiO_2 NPs/MPs heterojunction with 2D-Ti_3AlC_2 MAX cocatalyst for photocatalytic CO_2 reduction to CO/CH_4 in fixed-bed and monolith photoreactors. *J Mater Sci Technol* **106**, 195-210 (2022).

21. Fu G*, et al.* Rh/Al Nanoantenna Photothermal Catalyst for Wide-Spectrum Solar-Driven CO_2 Methanation with Nearly 100% Selectivity. *Nano Lett* **21**, 8824-8830 (2021).
