# Supplementary material for: Artificial cellulosic leaf with adjustable enzymatic CO2 sequestration capability
Source: Nat Commun. 2024 Jun 8;15:4898. doi: 10.1038/s41467-024-49320-y (PMC11162438; doi:10.1038/s41467-024-49320-y)
Supplement: Supplementary file 2 — Reporting Summary [file 41467_2024_49320_MOESM2_ESM.pdf]

nature portfolio

Corresponding author(s): Xing Zhu; Bin He

Last updated by author(s): 2024-5-31

Reporting Summary

Nature Portfolio wishes to improve the reproducibility of the work that we publish. This form provides structure for consistency and transparency in reporting. For further information on Nature Portfolio policies, see our Editorial Policies and the Editorial Policy Checklist.

Statistics

For all statistical analyses, confirm that the following items are present in the figure legend, table legend, main text, or Methods section.

n/a	Confirmed	
[ ]	[x]	The exact sample size (*n*) for each experimental group/condition, given as a discrete number and unit of measurement
[ ]	[x]	A statement on whether measurements were taken from distinct samples or whether the same sample was measured repeatedly
[ ]	[x]	The statistical test(s) used AND whether they are one- or two-sided<br>*Only common tests should be described solely by name; describe more complex techniques in the Methods section.*
[x]	[ ]	A description of all covariates tested
[x]	[ ]	A description of any assumptions or corrections, such as tests of normality and adjustment for multiple comparisons
[ ]	[x]	A full description of the statistical parameters including central tendency (e.g. means) or other basic estimates (e.g. regression coefficient) AND variation (e.g. standard deviation) or associated estimates of uncertainty (e.g. confidence intervals)
[x]	[ ]	For null hypothesis testing, the test statistic (e.g. *F*, *t*, *r*) with confidence intervals, effect sizes, degrees of freedom and *P* value noted<br>*Give P values as exact values whenever suitable.*
[x]	[ ]	For Bayesian analysis, information on the choice of priors and Markov chain Monte Carlo settings
[x]	[ ]	For hierarchical and complex designs, identification of the appropriate level for tests and full reporting of outcomes
[x]	[ ]	Estimates of effect sizes (e.g. Cohen's *d*, Pearson's *r*), indicating how they were calculated

Our web collection on statistics for biologists contains articles on many of the points above.

Software and code

Policy information about availability of computer code

Data collection	The experimental data in this study were obtained from domestic and foreign high-precision professional instrumentation, with high generalizability and accuracy.
Data analysis	We use professional data analysis software origin to analyze and graph the test data to ensure the reliability.

For manuscripts utilizing custom algorithms or software that are central to the research but not yet described in published literature, software must be made available to editors and reviewers. We strongly encourage code deposition in a community repository (e.g. GitHub). See the Nature Portfolio guidelines for submitting code & software for further information.

Data

Policy information about availability of data

All manuscripts must include a data availability statement. This statement should provide the following information, where applicable:

- Accession codes, unique identifiers, or web links for publicly available datasets
- A description of any restrictions on data availability
- For clinical datasets or third party data, please ensure that the statement adheres to our policy

Source data are provided with the paper. All data underlying this study are available from the corresponding author upon request.

Research involving human participants, their data, or biological material

Policy information about studies with human participants or human data. See also policy information about sex, gender (identity/presentation), and sexual orientation and race, ethnicity and racism.

Reporting on sex and gender	Not covered in this study.
Reporting on race, ethnicity, or other socially relevant groupings	Not covered in this study.
Population characteristics	Not covered in this study.
Recruitment	Not covered in this study.
Ethics oversight	Not covered in this study.

Note that full information on the approval of the study protocol must also be provided in the manuscript.

Field-specific reporting

Please select the one below that is the best fit for your research. If you are not sure, read the appropriate sections before making your selection.

☐ Life sciences ☐ Behavioural & social sciences ☒ Ecological, evolutionary & environmental sciences

For a reference copy of the document with all sections, see nature.com/documents/nr-reporting-summary-flat.pdf

Ecological, evolutionary & environmental sciences study design

All studies must disclose on these points even when the disclosure is negative.

Study description	Developing artificial leaves to address the environmental burden of CO2 is pivotal for advancing our Net Zero Future. In this study, we introduce EcoLeaf, an artificial leaf that closely mimics the characteristics of natural leaves. It harnesses visible light as its sole energy source and orchestrates the controlled expansion and contraction of “stomata” and the exchange of “petiole” materials to govern the rate of CO2 sequestration from the atmosphere. Furthermore, EcoLeaf, bearing a cellulose component akin to natural leaves, seamlessly integrates into ecosystem degradation and nutrient cycling processes. We propose that the carbon sequestration pathway within EcoLeaf is adaptable and can serve as a versatile biomimetic platform for diverse biogenic carbon sequestration pathways in the future.
Research sample	The leaf samples involved in this study were obtained from natural plants(Trachycarpus fortune, Cercis chinensis bunge, Pyrus ussuriensis, Zamioculcas zamiifolia engl, Holly) in nature.
Sampling strategy	Parallel control experiments were conducted by extracting leaves from different species and different parts of the same species to ensure the breadth and reproducibility of the experimental data obtained.
Data collection	During the entire research process, we collected data through the experimental procedures and survey methods described in the paper. The reaction conditions and data collection process have been detailed in the report to ensure the accuracy and reliability of the data.
Timing and spatial scale	*Indicate the start and stop dates of data collection, noting the frequency and periodicity of sampling and providing a rationale for these choices. If there is a gap between collection periods, state the dates for each sample cohort. Specify the spatial scale from which the data are taken*
Data exclusions	Not covered in this study.
Reproducibility	In order to guarantee the reproducibility and verifiability of the data, we have implemented a series of measures. These measures include documenting the detailed steps and procedures of the experiments or surveys, ensuring that other researchers can replicate our experiments or surveys. In the report, we also discuss potential data biases and provide corrective measures. We transparently report any limitations or uncertainties to enable others to evaluate our research methods and data processing procedures. Finally, we commit to following the guidelines of the Nature Reporting Summary to ensure that our data reliability statement complies with its specifications and requirements. We are dedicated to providing detailed, transparent, and accurate data reliability statements to promote the reproducibility and transparency of scientific research.
Randomization	Not covered in this study.
Blinding	*Describe the extent of blinding used during data acquisition and analysis. If blinding was not possible, describe why OR explain why blinding was not relevant to your study.*

Did the study involve field work? ☐ Yes ☒ No

Reporting for specific materials, systems and methods

We require information from authors about some types of materials, experimental systems and methods used in many studies. Here, indicate whether each material, system or method listed is relevant to your study. If you are not sure if a list item applies to your research, read the appropriate section before selecting a response.

Materials & experimental systems

n/a	Involved in the study	
☒	☐	Antibodies
☒	☐	Eukaryotic cell lines
☒	☐	Palaeontology and archaeology
☒	☐	Animals and other organisms
☒	☐	Clinical data
☒	☐	Dual use research of concern
☐	☒	Plants

Methods

n/a	Involved in the study	
☒	☐	ChIP-seq
☒	☐	Flow cytometry
☒	☐	MRI-based neuroimaging

Dual use research of concern

Policy information about dual use research of concern

Hazards

Could the accidental, deliberate or reckless misuse of agents or technologies generated in the work, or the application of information presented in the manuscript, pose a threat to:

No	Yes	
☒	☐	Public health
☒	☐	National security
☒	☐	Crops and/or livestock
☒	☐	Ecosystems
☒	☐	Any other significant area

Experiments of concern

Does the work involve any of these experiments of concern:

No	Yes	
☒	☐	Demonstrate how to render a vaccine ineffective
☒	☐	Confer resistance to therapeutically useful antibiotics or antiviral agents
☒	☐	Enhance the virulence of a pathogen or render a nonpathogen virulent
☒	☐	Increase transmissibility of a pathogen
☒	☐	Alter the host range of a pathogen
☒	☐	Enable evasion of diagnostic/detection modalities
☒	☐	Enable the weaponization of a biological agent or toxin
☒	☐	Any other potentially harmful combination of experiments and agents

Plants

Seed stocks	Not covered in this study.
Novel plant genotypes	Not covered in this study.
Authentication	Not covered in this study.
